# Olive (*Olea europaea* L.) Biophenols: A Nutriceutical against Oxidative Stress in SH-SY5Y Cells

**DOI:** 10.3390/molecules22111858

**Published:** 2017-10-29

**Authors:** Syed Haris Omar, Philip G. Kerr, Christopher J. Scott, Adam S. Hamlin, Hassan K. Obied

**Affiliations:** 1 School of Biomedical Sciences, Charles Sturt University, Wagga Wagga, NSW 2678, Australia; pkerr@csu.edu.au (P.G.K.); chscott@csu.edu.au (C.J.S.); hobied@csu.edu.au (H.K.O.); 2 Graham Centre for Agricultural Innovation, Charles Sturt University, Wagga Wagga, NSW 2678, Australia; 3 School of Science & Technology, University of New England, Armidale, NSW 2351, Australia; ahamlin@une.edu.au

**Keywords:** olive biophenols, oxidative stress, free radical scavenging, SH-SY5Y cells, Alzheimer’s disease

## Abstract

Plant biophenols have been shown to be effective in the modulation of Alzheimer’s disease (AD) pathology resulting from free radical-induced oxidative stress and imbalance of the redox chemistry of transition metal ions (e.g., iron and copper). On the basis of earlier reported pharmacological activities, olive biophenols would also be expected to have anti-Alzheimer’s activity. In the present study, the antioxidant activity of individual olive biophenols (viz. caffeic acid, hydroxytyrosol, oleuropein, verbascoside, quercetin, rutin and luteolin) were evaluated using superoxide radical scavenging activity (SOR), hydrogen peroxide (H_2_O_2_) scavenging activity, and ferric reducing ability of plasma (FRAP) assays. The identification and antioxidant activities in four commercial olive extracts—Olive leaf extract^TM^ (OLE), Olive fruit extract^TM^ (OFE), Hydroxytyrosol Extreme^TM^ (HTE), and Olivenol plus^TM^ (OLP)—were evaluated using an on-line HPLC-ABTS^•+^ assay, and HPLC-DAD-MS analysis. Oleuropein and hydroxytyrosol were the predominant biophenols in all the extracts. Among the single compounds examined, quercetin (EC_50_: 93.97 μM) and verbascoside (EC_50_: 0.66 mM) were the most potent SOR and H_2_O_2_ scavengers respectively. However, OLE and HTE were the highest SOR (EC_50_: 1.89 μg/mL) and H_2_O_2_ (EC_50_: 115.8 μg/mL) scavengers among the biophenol extracts. The neuroprotection of the biophenols was evaluated against H_2_O_2_-induced oxidative stress and copper (Cu)-induced toxicity in neuroblastoma (SH-SY5Y) cells. The highest neuroprotection values (98% and 92%) against H_2_O_2_-induced and Cu-induced toxicities were shown by the commercial extract HTE^TM^. These were followed by the individual biophenols, caffeic acid (77% and 64%) and verbascoside (71% and 72%). Our results suggest that olive biophenols potentially serve as agents for the prevention of neurodegenerative diseases such as AD, and other neurodegenerative ailments that are caused by oxidative stress.

## 1. Introduction

Alzheimer’s disease (AD), is one of the most frequent forms of neurodegenerative disorder associated with dementia in the elderly, and is believed to be caused by an abnormal deposition of amyloid-β peptide (Aβ) [1]. In normal cellular metabolism, reactive oxygen species (ROS) are produced, and include superoxide anion (O_2_^−^), hydroxyl (HO^•^), both of which are free radical species, and hydrogen peroxide (H_2_O_2_), which is a non-radical ROS. Excessive production of ROS appears to promote the redox imbalance, resulting in oxidative stress that has been implicated in Aβ-induced neurotoxicity in AD [2]. Evidence suggests that the production of ROS supports the “oxidative stress hypothesis” of AD [3]. In addition, metal ions induce the Aβ itself to produce the ROS, in turn causing oxidative stress-induced neurotoxicity [4]. Metal ions, such as iron and copper, that promote the formation of free radicals via the Fenton reaction, were observed to increase neurotoxicity in AD [5]. Importantly, these elevated markers for oxidative stress precede Aβ deposition and neurofibrillary tangles, suggesting that oxidative stress is an “early event” in AD pathogenesis. The endogenous antioxidant defense system is based on both enzymatic (superoxide dismutase, catalase, and glutathione peroxidase) reactions and non-enzymatic reactions involving compounds such as vitamins C and E, β-carotene, uric acid, and glutathione. These are found within the cytoplasm and various cell organelles to counter free radicals [6].

Apart from the aforementioned endogenous antioxidants, the secondary metabolites of plants, known as biophenols, have received considerable attention as exogenous antioxidants in the last two decades [7]. It has been proposed that the consumption of fruit and vegetable juices, containing high concentrations of biophenols, at least three times per week may delay the onset of AD [8].

A number of studies have reported the daily biophenol consumption in a number of European countries, including Finland (863 ± 415 mg/day) [9], France (1193 ± 510 mg/day) [10], Spain (820 ± 323 mg/day) [11], and Poland (1756.5 ± 695.8 mg/day) [12]. A Japanese study reported biophenol consumption at 961 ± 452 mg/day in an elderly population [13]. All these studies used the Phenol-Explorer database [14] or similar sources containing quantitative values measured by HPLC methods [15].

Indeed, increasing evidence suggests that the non-vitamin antioxidants, such as the biophenols, which show pleiotropic activity, including ROS scavenging and chelation of transition metals, especially iron and copper, are currently attracting considerable interest as potential therapeutic agents to counteract diseases, including AD, that are associated with oxidative stress [16].

Olive (*Olea europaea* L.) biophenols are an important component of the Mediterranean diet, and have received much attention due to their antioxidant effects [17]. A number of studies have suggested that the consumption of a traditional Mediterranean diet is associated with the reduction in the incidence of cardiovascular diseases, certain cancers and improved cognition [18,19,20]. The main phenolic compounds found in olive fruit are hydroxytyrosol (**3**) and tyrosol (**2**) respectively [21], while oleuropein (**4**) represents the major biophenol in olive leaf [22], followed by other constituents verbascoside (**6**), luteolin-7-*O*-glucoside, apigenin-7-*O*-glucoside, and tyrosol (**2**) [23].

The antioxidant activities of various olive preparations, both in vitro and in vivo, have been attributed to the biophenols, principally oleuropein (OL), hydroxytyrosol (HT), oleuropein aglycone (OA) and verbascoside (VB) [17]. Moreover, the activities of OL and HT were found to be higher than the well-known antioxidants, vitamins E and C. Perhaps surprisingly, tyrosol is reported as displaying neither antioxidant nor pro-oxidant activity [24]. Olive leaf extract also showed higher antioxidant activity than either vitamin C or vitamin E [25].

A limited number of cell line studies have reported the antioxidant activity of olive biophenols [26,27,28]. To the best of our knowledge, no study investigating the neuroprotective activity of olive biophenols in neuroblastoma (SH-SY5Y) cells has been carried out. As part of our continuing efforts at developing a dietary biophenols-based strategy targeting oxidative stress-induced cellular toxicity, we submit the present study to elucidate further the antioxidant capacity of olive biophenols (individually and as extracts) in cell-free as well as in an in vitro model of AD in SH-SY5Y cells. Accordingly, we determined the total phenols content (TPC) and total flavonoids content (TFC) of four commercial olive preparations.

## 2. Materials and Methods

### 2.1. Chemicals and Plant Extracts

Oleuropein (**4**), hydroxytyrosol (**3**), verbascoside (**6**) and luteolin (LU) (**8**) were purchased from Extrasynthese (Genay, France). Caffeic acid (CA) (**1**), quercetin (QU) (**9**) and rutin (RU) (**10**) were purchased from Sigma-Aldrich (Casstle Hill, Australia). Four commercial preparations were purchased, viz., Olive Leaf Extract^TM^ (OLE), equivalent to fresh leaf 1 g/mL or oleuropein 4.4 mg/mL from Comvita^TM^ (Brisbane, Australia); Olive Fruit extract^TM^ (OFE), each mL stated to contain 5 mg of oleuropein, from Nature Goodness^TM^ (Smeaton Grange, Australia); Hydroxytyrosol Extreme^TM^ (HTE), each 100 mg olive leaf extract capsule stated to provide 25 mg of hydroxytyrosol, from ProHealth^®^ (Carpinteria, CA, USA); and 200 mg of Olivenol Plus^TM^ capsules (OLP), made with 12 mg (6%) of HIDROX^®^, a patented formula of HT derived from olive juice, from CREAGRI^TM^ (Hayward, CA, USA). Nitrotetrazolium blue chloride (NBT), monobasic potassium phosphate, phenol red (ACS reagent), reduced β-nicotinamide adenine dinucleotide disodium salt (NADH), phenazine methosulfate, horseradish peroxidase (HRP) (type VI), and 3-(4,5-dimethylthiazol-2-yl)-2,5-diphenyltetrazolium bromide (MTT), hydrogen peroxide (H_2_O_2_), sodium acetate trihydrate, hydrochloric acid and iron (III) chloride hexahydrate, 2,4,6-tripyridyl-*s*-triazine (TPTZ), Trolox^TM^, Neuroblastoma cell line (SH-SY5Y), Dulbecco’s modified Eagle’s medium (DMEM), fetal calf serum (FCS), non-essential amino acid solution (NEAA), Minimum essential medium (MEM), l-glutamine solution, penicillin-streptomycin solution, trypsin-EDTA solution, trypan blue, dimethyl sulfoxide (DMSO), Tris-HCl buffer were purchased from Sigma Aldrich (Australia).

### 2.2. Sample Preparation

**Figure 1:**
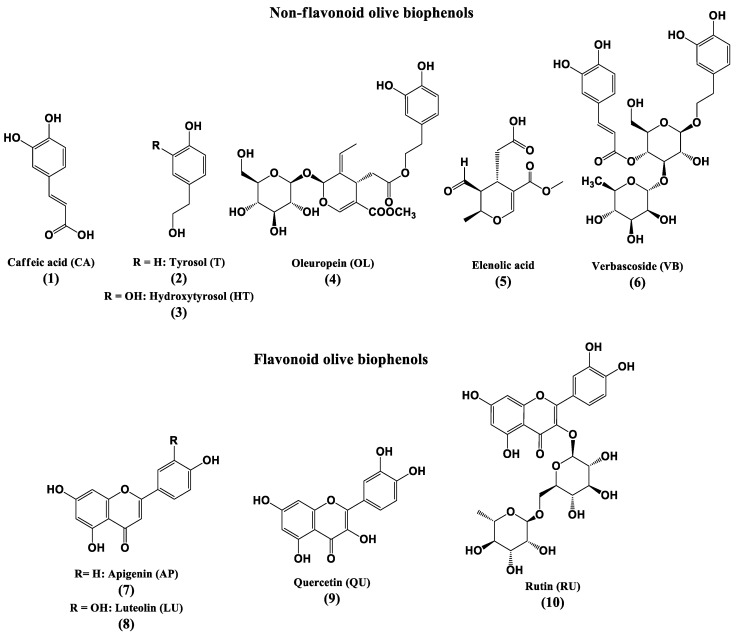
Structures of the major non-flavonoid and flavonoid olive biophenols.

All standard non-flavonoid biophenols (CA, HT, OL and VB), flavonoid biophenols (LU, QU and RU) (Figure 1) and the commercial olive extracts (OLE, OFE, OLP and HTE) were prepared in 50% methanol as stock solutions followed by ultra-sonication and filtration (nylon syringe filter 0.25 μm) before each assay and consumed within 4 h of preparation to minimize air-oxidation.

### 2.3. Determination of Total Phenol Content (TPC) in Commercial Extracts

TPC in the commercial olive extracts (OLE, OFE, HTE and OLP) was determined as previously described [29]. Aliquots (100 μL) of various concentrations of olive extract or a blank (methanol: water, 1:1, *v*/*v*) were added to 10 mL volumetric flasks each containing 7–8 mL of water. Folin-Ciocalteu reagent (500 μL) was added, followed after one min by Na_2_CO_3_ solution (1.5 mL, 20% *w*/*v*). Immediately after gentle shaking, the flasks were made to volume with water and incubated for one hour at 25 °C followed by recording the absorbance at 760 nm. All samples were analyzed in triplicate. The total phenolic compounds in the extract in gallic acid equivalents (GAE) were calculated by the following formula:

T=C×V÷M

where *T* is the total phenolic contents, mg/g of extract, in GAE; *C* is the concentration of gallic acid established from the calibration curve, mg/mL; *V* is the volume of the extract, mL; *M* is the weight of the extract (mg).

### 2.4. Determination of Total Flavonoid Content (TFC) in Commercial Extracts

TFC content in the olive extracts was determined according to the AlCl_3_ colorimetric assay [30] with minor modifications. In 3 mL of 80% methanol, 500 μL of each olive extract or quercetin was added to a 10 mL volumetric flask followed by 300 μL of NaNO_2_ (5% *w*/*v*). After 5 min, 300 μL of AlCl_3_·6H_2_O (10% *v*/*v*) was added, followed by NaOH (1 M; 2 mL). The reaction mixture was shaken until homogeneous before the volume was made up to 10 mL with water. After gentle mixing, the absorbance was read against a blank solution at 510 nm. Total flavonoid content was expressed as milligrams of quercetin equivalent per gram dry weight of air-dried material (mg·QE/g). Each extract was analyzed in triplicate.

### 2.5. HPLC-DAD-Online ABTS Radical Scavenging Analysis

The HPLC analysis was performed according to the earlier described method [31] with slight modifications using a Varian Prostar 240 solvent delivery system equipped with a Varian Prostar 335 diode array detector (DAD), a Varian Prostar 410 auto-sampler along with Star Chromatography workstation version 6.41 (Varian, Australia) controller. A flow rate of 0.7 mL/min and an injection volume of 10 μL were used.

The outflow from the DAD was connected to a reaction coil through a T-intersection. ABTS^•+^ (diluted from a stock solution of 3 mM to give an absorbance = 0.70 at 734 nm) was pumped to the reaction coil through the T-intersection by a Perkin-Elmer series 10 HPLC pump. A PEEK reaction coil, 3.4 m × 0.178 mm id, was maintained at 37 ± 1 °C in a Varian HPLC column temperature controller. Detection of ABTS^•+^ absorbance was monitored at 414 nm by a Varian 9050 UV-Vis detector (Santa Clara, CA, USA). Data collected from this arrangement generated positive peaks by reversing the polarity of the analogue signal.

Sample analysis was performed by gradient elution from a 250 mm × 4.6 mm id, 5 μM, Gemini C-18 column (Phenomenex, Sydney, Australia) with a Security Guard (Phenomenex) guard cartridge. The mobile phases were freshly prepared, degassed under vacuum filtration using Phenomenex nylon 0.45 μm membranes, and placed in a Sanophon ultrasonic bath (Ultrasonic Industries Pty. Ltd., Sydney, Australia) with the rated power output of 375 W for 15 min prior to HPLC analysis.

Solvent A was a mixture of water/formic acid (1000:3, *v*/*v*), whereas solvent B was acetonitrile/water/formic acid (600:400:3, *v*/*v*/*v*). A flow rate of 0.7 mL/min, injection volume of 5 μL and gradient elution for total run time of 30 min were used as follows: initial conditions 90% solvent A; solvent B was increased to 25% over 2 min; then, solvent B was increased to 45% over 3 min, followed by isocratic elution for 2 min. Solvent B was increased to 80% over 13 min and to 100% over 5 min and back to initial composition in 5 min. The system was allowed to equilibrate for 20 min between runs.

### 2.6. Liquid Chromatography-Mass Spectrometry (LC-MS)

Samples were analyzed by an Agilent 1200 series liquid chromatograph (Agilent Technologies, Waldbronn, Germany) by gradient elution on a 150 mm × 4.6 mm id, 3 μm, Phenomenex C-18 column (Lane Cove, Australia). The separation conditions as for HPLC-DAD were maintained. However, the DAD was set to record chromatograms at 280 and 325 nm.

The outflow from the DAD was connected to a Agilent 6410 triple-quadrupole mass analyzer (Agilent Technologies, Santa Clara) equipped with an electrospray ionization interface. MS analysis was performed in the negative ion mode (*m*/*z* 100–1500) using nitrogen gas under the following conditions: gas temperature, 300 °C; gas flow rate, 12 L/min; nebulizer pressure, 45 psi; capillary voltage, 4 kV; cone voltage, 100 V.

Data were analyzed using Mass Hunter Workstation version B.04.00 (Agilent Technologies).

### 2.7. Superoxide Radical (SOR) Scavenging Assay

The method for the determination of SOR scavenging activity was adapted, with modification, from a previous report [27]. Fifty μL of different concentrations of olive biophenols, 50 μL of NBT (200 μM), 50 μL of NADH (624 μM), and 50 μL of phenazine methosulfate (80 μM) were added sequentially. The reaction mixture was incubated at room temperature for 5 min, and the absorbance at 560 nm was read using a VersaMax^TM^, tunable automated microplate reader (Molecular Devices, Sunnyvale, CA, USA).

Phosphate buffer was used as a negative control. Due to its instability, fresh phenazine methosulfate solution was prepared for each set of experiments [27]. The scavenging percentage was calculated as

$$Scavenging\;\%=[1-\frac{absorbance\;of\;sample\;at\;560\;nm}{absorbance\;of\;control\;at\;560\;nm}]\times 100$$

### 2.8. In Vitro H_2_O_2_ Scavenging Assay

The H_2_O_2_ scavenging assay used was described previously [27]. Fifty μL of freshly prepared 2 mM H_2_O_2_ solution, as per Beers and Sizer (1952) was mixed with 50 μL of different concentrations of olive biophenols. The reaction mixture was incubated at room temperature (20 ± 2 °C) for 20 min. The mixture (100 μL) made from freshly prepared HRP (0.3 mg/mL) and phenol red (4.5 mM) in 0.1 M phosphate buffer was added to the reaction mixture containing biophenols and H_2_O_2_. After 10 min of incubation at room temperature, the absorbance was measured at 610 nm using the Versamax^TM^ microplate reader.

All the biophenol samples were prepared in 50% methanol, while the reagents were prepared in 0.1 M phosphate buffer (pH 7.4). The scavenging percentage was calculated as

$$Scavenging\;\%=[1-\frac{absorbance\;of\;sample\;at\;610\;nm}{absorbance\;of\;control\;at\;610\;nm}]\times 100$$

### 2.9. Ferric Reducing/Antioxidant Power (FRAP) Assay

The FRAP assay procedure was conducted according to earlier described methods [31,32] with slight modification. The working FRAP reagent was prepared by mixing three stock reagent solutions including 300 mM acetate buffer (pH 3.6, 25 mL), 5 mM TPTZ in 40 mM HCl (2.5 mL) and 5 mM FeCl_3_·6H_2_O (2.5 mL). The mixture was warmed at 37 °C prior to use.

For each sample, 200 μL FRAP reagent was added, with 10 μL of olive biophenols and 40 μL of deionized water. After incubation at 37 °C (water bath) the absorbance at 593 nm was measured using a Fluostar omega plate reader (BMG-Labtech, Ortenberg, Germany). The final result was expressed as the concentration of antioxidant having a ferric reducing ability for 1 g of sample (mM/g). A higher absorbance of the reaction mixture indicated greater reducing power. Trolox^TM^ was used as standard and the total reducing power was expressed as Trolox^TM^ equivalents. Samples were analyzed in triplicate.

### 2.10. Cell Culture

The neuroblastoma (SH-SY5Y) cells were grown in a clear sterile T-75 flask with the medium (manufacturer protocol) 50% Minimum Essential Media (MEM) and 50% Ham’s F-12 supplemented with 15% inactivated fetal calf serum, 1% of 100 units/mL penicillin/streptomycin, 1% l-glutamine and 1% NEAA, followed by incubation at 37 °C under 5% CO_2_/95% humidified air in an incubator. After reaching 80–90% confluency, the cells were passaged usually every third day by using trypsin-EDTA solution, the cells were detached the T-75 flask, and subsequently cultured in the fresh medium. Viable and dead cells were quantified using a hemocytometer following the addition of 10% Trypan Blue.

### 2.11. H_2_O_2_-Induced Toxicity in SH-SY5Y Cells and Neuroprotective Potential of Olive Biophenols

In order to determine the LD_50_ value of H_2_O_2_ against SH-SY5Y cells, eleven dilutions of the stock H_2_O_2_ (30%) were freshly prepared and incubated with the SH-SY5Y cells at an initial density 5 × 10^3^ cells per well in clear sterile 96 well-plates followed by incubation at 37 °C under 5% CO_2_/95% humidified air in an incubator.

The olive biophenols were prepared at various concentrations and incubated with cells at a density of 5 × 10^3^ cells/well in the sterile clear 96 well plates and maintained at 37 °C under 5% CO_2_/95% humidified air in an incubator for 24 h [33]. To determine the protective effect of the olive biophenols, freshly prepared H_2_O_2_ (at the LD_50_ concentration) was added to each well containing cells pre-treated with olive biophenols, and incubated for further 24 h at 37 °C under 5% CO_2_/95% humidified air in an incubator.

### 2.12. Copper (Cu)-Induced SH-SY5Y Cells Toxicity and Olive Biophenols Treatment

The copper-induced SH-SY5Y cells toxicity assay was conducted according to the previously described method [34] with slight modifications. The SH-SY5Y cells were seeded in clear sterile 96-well plates at a density of 5 × 10^3^ cells per well and incubated at 37 °C under 5% CO_2_/95% humidified air for 24 h. In order to determine the LD_50_ of copper in SH-SY5Y cells, various concentrations (10–500 μM) of Cu were treated with the SH-SY5Y cells and incubated for 24 h under the same conditions.

The olive biophenols were prepared at various concentrations and incubated with cells at a density of 5 × 10^3^ cells/well in the sterile clear 96 well plates and maintained at 37 °C under 5% CO_2_/95% humidified air in an incubator for 24 h. The protective effect of olive biophenols were determined by adding Cu (EC_50_ value) to the pre-treated SH-SY5Y cells with or without various concentrations of olive biophenols followed by incubation for 24 h.

### 2.13. Cell Viability Assay

Cell viability was determined by MTT assay. The cells were treated with 10 μL of MTT (5 mg/mL) in phosphate buffered saline (pH 7.4) to the each well followed by incubation for 4 h at 37 °C [35]. The formazan crystals were generated by viable mitochondrial succinate dehydrogenase from MTT. The supernatant were then aspirated off and the formazan crystals were dissolved in 50 μL of DMSO. The absorbance was measured at 570 nm after 15 min, using the Omega Star micro plate reader [36]. The cell viabilities were expressed as percentages of survival relative to the control sample.

### 2.14. Statistical Analysis

Statistical analyses were performed with one-way ANOVA test followed by a post-hoc analysis (Tukey’s multiple comparison test) and the EC_50_ values (the concentrations required to inhibit the 50% of enzyme activity under the experimental conditions) were calculated by using GraphPad Prism version 5.0 software for Windows (GraphPad Software, Inc., San Diego, CA, USA). All values were presented as mean ± standard error of the mean (mean ± SEM) for each group. The “*p*” value *p* < 0.05 was considered significant.

## 3. Results and Discussion

### 3.1. Total Phenol and Total Flavonoid Contents

The TPC (expressed as mg GAE/g) of each of the commercial olive extracts was determined using Folin-Ciocalteu’s reagent and found to be as follows: HTE (574.47 ± 35.39) > OLP (30.12 ± 0.83) > OLE (7.87 ± 0.05) > OFE (4.64 ± 0.09). An in vitro study has shown that the maximum optimization yield of total biophenols content determined by the Folin-Ciocalteu assay was 250.2 mg GAE per 100 mg dry weight of olive leaf extract [37]. Our results show more than double the amount of total phenols in HTE, with the other extracts being substantially lower.

The flavonoid concentrations in the various olive extracts in the range from 24.17 to 823.12 mg quercetin equivalent (QE) per gram. The contents were, HTE (823.12 ± 12) followed by OLP (174.70 ± 1.72), OLE (32.03 ± 0.73), and least in OFE (24.17 ± 0.33). Again the commercial product, HTE, is the front-runner with the highest flavonoid content, regardless of sampling parameters (olive cultivar, leaf age or sampling date).

### 3.2. Identification and Antioxidant Profile of Commercial Olive Extracts

**Figure 2:**
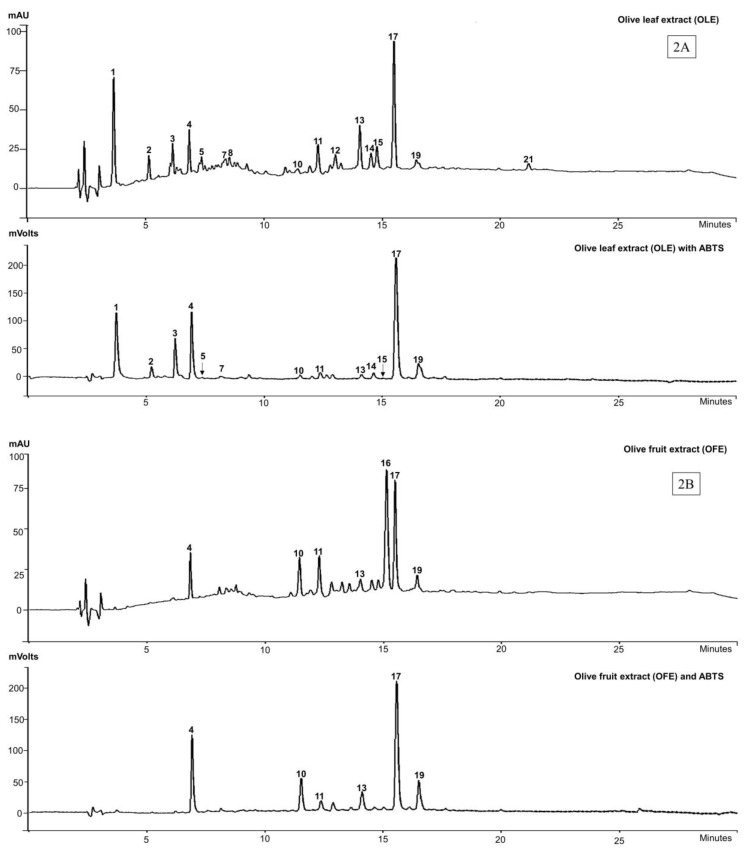

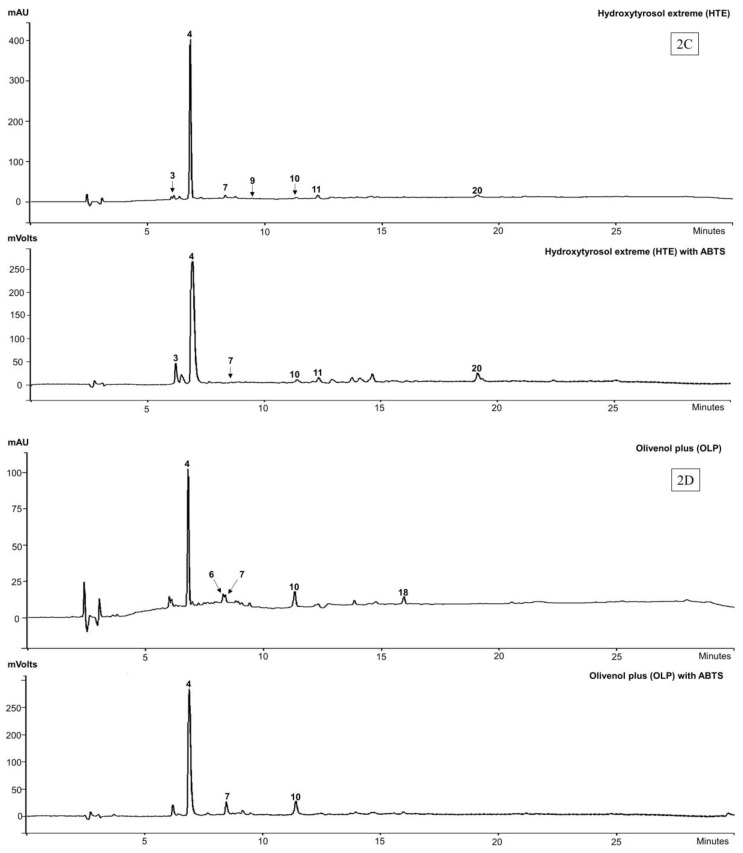
HPLC-DAD-ABTS chromatograms of four commercial extracts of olive at 280 nm and ABTS scavenging at 414 nm. (**2A**) olive leaf extracts (OLE); (**2B**) olive fruit extracts (OFE); (**2C**) Hydroxytyrosol Extreme^TM^ (HTE) and (**2D**) Olivenol Plus^TM^ (OLP).

The phenolic composition along with antioxidant profile of all the commercial olive extracts were assessed by HPLC-DAD, online-ABTS scavenging activity chromatograms and confirmed by LC-MS. Due to the characteristic absorption of secoiridoids at 240 nm, which are abundant in the *Oleaceae* family [38], the wavelength 280 nm was chosen to allow for the detection of a wide range of phenolic compounds. The online-ABTS scavenging activities of each extract measured at 414 nm are shown in Figure 2. Twenty-one peaks appeared in the HPLC chromatograms of the olive extracts and yielded seventeen identifiable compounds, with four compounds (peaks 1, 2, 16 and 21) remaining unidentified (Table 1; Figure 2).

The major biophenols identified in the OLE were: hydroxytyrosol (**3**) (peak 4), oleuropein agylcone-1 (peak 7), elenolic acid (**5**) (peak 8), verbascoside (**6**) (peak 10), luteolin-7-*O*-glucoside (peak 11), flavonoid glucosides (peaks 14 and 15) and oleuropein (**4**) (peak 17) as the major constituent. Other studies [39,40] show biophenol profiles of OLE that are similar to those we report here, also with oleuropein (**4**) as the dominant compound.

For the OFE, the biophenols were the same those of the olive leaf extracts except for the tentatively identified oleuropein agylcone-1 (peak 7) and flavonoid glycosides (peaks 14 and 15). The two compounds, found at peaks 12 and 13 in OLE (Figure 2A) were partially characterised as being secoiridoids (λ_max_, 240–280) but not assigned particular identities, while peak 13 was also apparent in OFE (Figure 2B).

In HTE, the identified components were hydroxytyrosol glucoside (peak 3), with hydroxytyrosol (**3**) (peak 4) appearing as the major component, while oleuropein agylcone-1 (peak 7), elenolic acid glucoside (peak 9), verbascoside (**6**) (peak 10), luteolin-7-*O*-glucoside (peak 11) and oleuropein aglycone-2 derivative (peak 20) appear as minor components of the extract.

The biophenol profile for OLP was similar to that of the HTE extract except for hydroxytyrosol glucoside (peak 3). Tyrosol (**2**) (peak 6) was found only in the OLP extract, but does not appear in the MS analysis, because of its acidic character and the operating parameters (solvent and MS conditions) being unsuitable for its ionization [41]. There was a compound, tentatively identified as a non-phenolic secoiridoid derivative (peak 18) appearing in OLP (Figure 2D) but with a λ_max_ at 320 nm.

The HPLC/ABTS scavenging activity results suggest that the antioxidant activity of OLE resides predominantly in 14 of the compounds corresponding with peaks 1 to 21 (excepting peaks 6, 8, 9, 12, 16, 18 and 21) of the chromatogram. The compounds identified in OFE that show ABTS scavenging activity are those corresponding to hydroxytyrosol (**2**), verbascoside (**5**), luteolin-7-*O*-glucoside, unknown “secoiridoid”, oleuropein (**4**), and oleuroside (peaks 4, 10, 11, 13, 17 and 19 respectively). The large peak (16) at retention time (T_R_) 15 min is an unknown compound that does not appear in any of the other extracts (Figure 2B). Nor does it appear to exhibit any ABTS scavenging activity.

All the major compounds identified in HTE, viz., hydroxytyrosol glucoside, hydroxytyrosol (**3**) oleuropein agylcone-1, verbascoside (**6**), luteolin-7-*O*-glucoside and oleuropein aglycone-2 show ABTS scavenging activity, except the elenolic acid glucoside. In OLP, hydroxytyrosol (**3**), oleuropein agylcone, and verbascoside (**6**), at peaks 4, 7, and 10 respectively, exhibited ABTS scavenging activity except tyrosol (**1**) (peak 6) and the non-phenolic “secoiridoid” (peak 18).

We found that the biophenols, hydroxytyrosol (**3**) (peak 4) and verbascoside (**6**) (peak 10) were present in all the four commercial extracts (OLE, OFE, HTE and OLP) and showed strong online-ABTS scavenging activity (relative to peak heights in the HPLC chromatograms). Oleuropein aglycone-1 (peak 7) was found in three extracts (OLE, HTE and OLP). Luteolin-7-*O*-glucoside (peak 11) was also detected in three (OLE, OFE, HTE) of the 4 the extracts, albeit in very variable amounts, while oleuropein (**4**) (peak 17) was dominant in OLE and OFE (Table 1).

Our HPLC analysis results of olive extracts were consistent with previously reported studies [25,48,49]. According to the manufacturers, the two commercial extracts, OLE and HTE, were prepared from olive leaf, while OFE and OLP were prepared from olive fruit pulp. Moreover, oleuropein (**4**) was the main constituent in OLE (4.4 mg/mL) and OFE (5 mg/mL), while hydroxytyrosol (**3**) serves as the primary constituent in HTE (25 mg/100 mg extract) and OLP (12 mg/capsule) extracts as stated by the manufacturers. Our results support the manufacturers’ claims.

### 3.3. Superoxide Radical (SOR) Scavenging Activities

The non-enzymatic phenazine methosulfate-NADH system generates SORs, which reduce NBT to a purple formazan dye. The generated SORs reduce the yellow, water-soluble NBT^2+^ cations to blue, water-insoluble diformazan. In an aqueous solution at physiological pH, phenols (e.g., as flavonoids) and/or their phenoxyl radicals can reduce SOR in a two-step process to form H_2_O_2_ [50]. Thus, the decrease in absorbance at 560 nm (indicative of less reduction of NBT) with olive biophenols indicates the consumption of superoxide anion in the reaction mixture. The lower the absorbance at 560 nm, the more potent an SOR scavenger is the sample (Table 2).

For the SOR scavenging activity of the non-flavonoid phenols, VB (**6**) was higher than OL (**4**), HT (**3**) and CA (**1**), and the corresponding concentrations that scavenge 50% of the SOR (EC_50_) values were 119.4, 258, 291.4 and 436.3 μM respectively. The highest activity for VB (**6**) was consistent with our lab’s earlier published results [27], and suggests the presence of the two catechol functions in its structure confers the greater antioxidant activity [51].

In the case of the flavonoid olive biophenols, QU (**9**) showed the maximum SOR scavenging activity followed by RU (**10**) and LU (**8**). The corresponding EC_50_ values were 83.71, 143.2 and 234 μM respectively (Table 2). The superiority of QU (**9**) over the other flavonoids (RU (**10**) and LU (**8**)) for SOR scavenging activity, was also indicated by earlier studies [52,53].

Each of these flavonoids possesses a catechol function attached to the B-ring, which is conjugated with the α,β-unsaturated carbonyl system of C-ring. This structural configuration is likely to confer stability of their radicals and in turn lead to greater SOR scavenging ability in comparison with the non-flavonoids. The presence of an additional hydroxyl group at the 3-position (C-ring) in the QU (**9**) molecule, might explain why it exhibited slightly more potent activity than RU (**10**) and LU (**8**). In sum, we found that the activities of the individual compounds were in the order: QU > VB > RU > LU > OL > HT > CA.

For the commercial olive extracts, OLE showed the maximum scavenging potency (Table 2). In contrast, at higher concentration (>50 μg/mL), OLE showed hormetic behavior (characterized by low dose stimulation and by high dose inhibitory or toxic effect) in the SOR scavenging activity [54]. Olive extracts can be ranked due to their SOR activity as follows: OLE > HTE > OLP > OFE. The results indicate that the extract having lowest total phenol content and total flavonoid content, has the lowest SOR scavenging activity as seen in OFE. Though HTE had the highest total phenol and total flavonoid content, it was less potent than OLE as an SOR scavenger.

### 3.4. H_2_O_2_ Radical Scavenging Activities by Olive Biophenols

Olive biophenols behave as good electron and hydrogen donors due to the presence of phenolic hydroxyl groups, and may thus accelerate the conversion of H_2_O_2_ into H_2_O. The unreacted H_2_O_2_ is consumed by HRP to oxidize phenol red, and the oxidation product absorbance is measured at 610 nm. The highest H_2_O_2_ scavenging activity was shown by VB with an EC_50_: 0.66 mM, which is consistent with our lab’s earlier published results [27], while the rest of the non-flavonoid olive biophenols show similar but lower potency (~1.0 mM) scavenging activities (Table 2). In contrast, none of the flavonoid olive biophenols showed H_2_O_2_ scavenging activity (Table 2). The H_2_O_2_ scavenging activity of HTE extract was higher than that of OLE, OFE, and OLP, with the corresponding EC_50_ values 115.8, 120.6, 217, 280.3 μg/mL respectively.

### 3.5. Ferric (Fe^3+^) Reducing Antioxidant Power Assay (FRAP)

The FRAP assay depends on the reduction of the colorless ferric complex (Fe^3+^ tripyridyltriazine) to the blue-colored ferrous complex (Fe^2+^ tripyridyltriazine) by the action of electron donating antioxidants at low pH [32]. The standard curve was prepared using different concentrations of Trolox^®^ ranging from 0 to 1200 μM, giving R^2^ = 0.9999 (data not shown). Olive biophenols showed significant reducing power in the FRAP assay (Table 2).

As with SOR and H_2_O_2_, VB showed the highest activity from among the non-flavonoid compounds. QU had highest activity followed by LU and RU among flavonoids. HTE showed the highest antioxidant activity from the extract group of olive biophenols followed by OLE. The “commercial extracts” can be ranked according to their reducing power in the following order HTE > OLE > OFE > OLP.

Iron plays an important role in AD pathology via the Fenton reaction in which Fe^2+^ reacts with H_2_O_2_ to produce the hydroxyl radical (OH). Studies show that iron accumulates in the same brain regions where the Aβ is deposited, i.e., the hippocampus, the parietal cortex, and the motor cortex [55,56]. Interestingly, accumulation and binding of Fe^3+^ to the tau protein precedes the aggregation of hyperphosphorylated tau followed by subsequent formation of neurofibrillary tangles [4,57]. The majority of dietary source (plants) non-haem iron enters the gastrointestinal tract as Fe^3+^, which is non-bioavailable. Fe^2+^ shows better absorption than Fe^3+^ because the latter precipitates out of solution at around pH 7, i.e., under normal physiological conditions. Olive biophenols may inhibit the intestinal absorption of Fe^2^^+^ and Fe^3+^ thus preventing the redox cycling of iron [58], suggesting decrease in oxidative stress. Furthermore, it may cause a decrease in the availability of Fe^3+^ binding to tau protein, which in turn leads to a decrease in further aggregation in the AD patient brain.

### 3.6. Neuroprotective Effect of Olive Biophenols against H_2_O_2_-Induced Cytotoxicity in SH-SY5Y Cells

**Figure 3:**
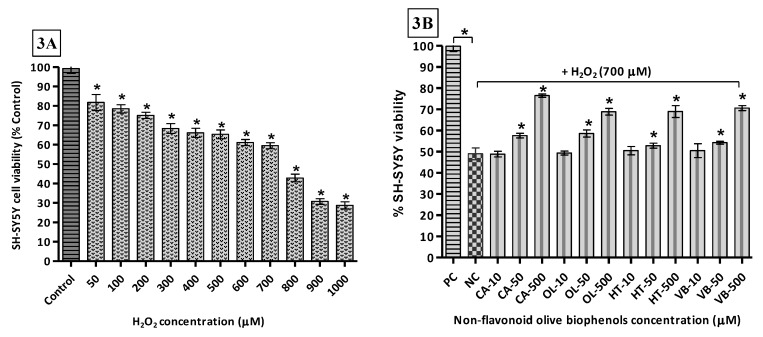

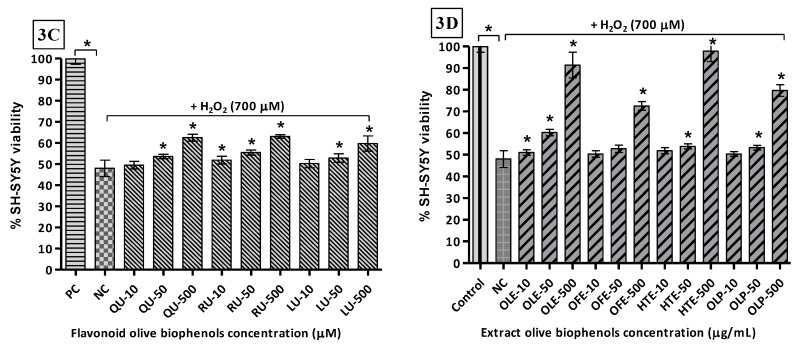
H_2_O_2_-induced SH-SY5Y cells toxicity and protection by olive biophenols: SH-SY5Y cells were treated with different concentrations of H_2_O_2_ for 24 h (**3A**). SH-SY5Y cells were incubated with different concentrations of non-flavonoid olive biophenols (**3B**), flavonoid olive biophenols (**3C**) and extract olive biophenols (**3D**) for 24 h followed by 700 μM of H_2_O_2_ for 24 h. The results are mean ± SE of each parallel measurements analyzed by one way ANOVA, * *p* < 0.05 vs. control and negative control. NS: non-significant. PC: positive control (cells with media), NC: negative control (cells and H_2_O_2_ without biophenols), CA: caffeic acid, OL: oleuropein, HT: hydoxytyrosol, VB: verbascoside, QU: quercetin, RU: rutin, LU: luteolin, OLE: olive leaf extract, OFE: olive fruit extract, HTE: Hydroxytyrosol extreme^TM^, OLP: Olivenol plus^TM^.

The toxicity of H_2_O_2_ towards SH-SY5Y cells was determined by exposing them to concentrations of the oxidant of between 0 and 1000 μM. The results indicate that the LD_50_ (H_2_O_2_) = 654.6 μM (Figure 3A). Thus, the concentration of H_2_O_2_ used (700 μM) in the neuroprotection experiments was to approximate the LD_50_. Following pre-treatment of the cells with the olive compounds/extracts, it was found that the non-flavonoid olive biophenol, CA, affords the maximum protection, with cell viability of 77% after 24 h of exposure to H_2_O_2_ (Figure 3B). This was followed by 71% viability with VB (Figure 3C), while OL and HT showed equal viabilities (69%) (Figure 3B). The flavonoid olive biophenols appear to be less protective, with QU and RU showing equal viabilities (63%), while treatment with LU reached 59% cell viability against H_2_O_2_ toxicity (Figure 3C).

Intuitively, perhaps, it is expected that the single compounds would afford the greatest levels of protection against cell damage. However, our results indicate otherwise. At the highest concentrations examined, the olive extract, HTE, affords the highest protection, with 98% cell viability, followed by OLE (92%), OLP (80%) and OFE (73%) (Figure 3D). It looks obvious that a synergic effect is operational because the mixtures of compounds in the extracts very likely contain concentrations of the antioxidants at considerably lower levels than was used in the single compound experiments reported here.

It is pertinent to note that H_2_O_2_ is a normal by-product of cellular metabolism and at low, micromolar, concentrations it is considered a signaling molecule that can modulate processes such as cell growth, differentiation, and migration [59], whereas at higher concentrations it can cause severe oxidative stress. As an oxidizing agent, H_2_O_2_ directly inactivates thiol (-SH) containing enzymes via oxidation to disulfide linkages [60]. Furthermore, due to absence of unpaired electrons in H_2_O_2_, unlike other ROS, it can readily cross biological membranes and react with metal ions (Fe^2+^ and Cu^2+^), generating highly toxic ROS [61].

In addition, studies show that H_2_O_2_ may activate the enzymes γ-secretase [62] and also β-secretase, which may play a role in the oxidative-stress-induced BACE1 expression in AD [63]. Our results suggest that each of the individual olive biophenols exhibits a significant neuroprotective effect. In the case of the commercial extracts, this is enhanced, presumably due to a synergic effect of the mixtures.

### 3.7. Neuroprotective Effect of Olive Biophenols against Copper-Induced Cytotoxicity in SH-SY5Y Cells

Studies have shown that copper (Cu) overload to human-derived lung, liver cells (A-549 and HepG2) [64] and neuroblastoma SH-SY5Y cells [65] significantly increased the concentration of this metal inside the cells in a dose-dependent manner. In addition, these Cu overloads and which is ultimately cause concomitant increase in ROS production and more specifically in H_2_O_2_, which are ultimately a risk factor for AD.

**Figure 4:**
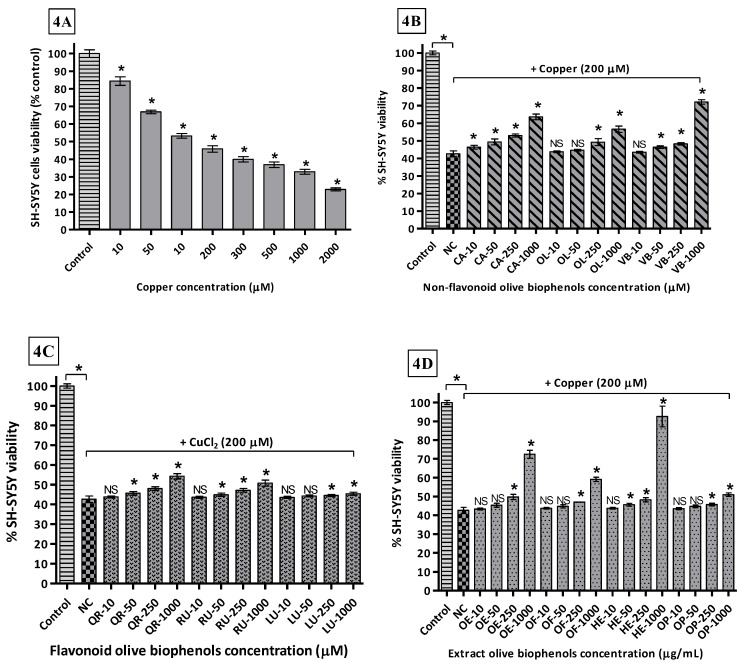
Copper-induced SH-SY5Y cells toxicity and protection by olive biophenols: SH-SY5Y cells were treated with different concentrations of copper for 24 h (**4A**). SH-SY5Y cells were incubated with different concentrations of non-flavonoid olive biophenols (**4B**), flavonoid olive biophenols (**4C**) and extract olive biophenols (**4D**) for 24 h followed by 200 μM of copper for 24 h. The results are mean ± S.E. of each parallel measurements analyzed by one way ANOVA (Tukey’s test), * *p* < 0.05 vs. control and negative control. NS: non-significant. PC: positive control (cells with media), NC: negative control (cells and H_2_O_2_ without biophenols), CA: caffeic acid, OL: oleuropein, HT: hydoxytyrosol, VB: verbascoside, QU: quercetin, RU: rutin, LU: luteolin, OLE: olive leaf extract, OFE: olive fruit extract, HTE: Hydroxytyrosol extreme^TM^, OLP: Olivenol plus^TM^.

By using different concentrations of Cu^2+^ incubated with SH-SY5Y cells after 24 h, determined the LD_50_ 169.2 μM causing the maximum toxicity of 77% compared to control (Figure 4A). In the non-flavonoid olive biophenols group, VB showed the highest protective activity 72% against Cu-induced SH-SY5Y toxicity (Figure 4B). CA showed the second highest protective activity 64% (Figure 4B), while OL and HT showed (Figure 4B) almost similar protective abilities 56% and 53% respectively. The flavonoids biophenols showed inferior protective activity to the non-flavonoids against Cu-induced SH-SY5Y cells. QR showed max protection of 54% followed by RU 51%, while LU was the least active flavonoid 44% (Figure 4C) against Cu-induced SH-SY5Y toxicity. Olive extracts showed significantly higher protective activity, where HTE was the most protective showing 92% of activity (Figure 4D). OLE was the second most protective showing 73% of protection (Figure 4D), while OFE showed only 59% of protection followed by the least active OLP 51% activity (Figure 4D).

The metal-ion-binding sites on Aβ provide a very promising target for the development of new therapeutics, where copper-treated SH-SY5Y cells may represent a model of copper overload in the brain leading to neurodegeneration of AD. The ongoing research in Cu-specific chelating agents, chaperones, or antioxidants are foci for the prevention of Cu mediated Aβ neurotoxicity and ROS production in AD by Cu-chelation therapy, which is an emerging trend in current research [66].

Our results suggest that olive biophenol extracts are superior to the individual olive non-flavonoids and flavonoids for protection of the SH-SY5Y cells against Cu-induced toxicity, which could be a promising therapy for the treatment of AD. Olive flavonoids exhibit relatively poor protection and the possible reason behind their poor activities is that they are susceptible to auto-oxidation and conversion into their *O*-methylated metabolites, with the lack of a hydroxyl group ultimately leading to slight cytotoxicity to the SH-SY5Y cells [67].

## 4. Conclusions

The present study is the first report of the phenolic composition and antioxidant activity comparison of four commercial olive extracts (two from leaf and two from fruit) with the individual biophenols in the SH-SY5Y cells. The individual olive biophenols, hydroxytyrosol (**3**) and verbascoside (**6**) were the most abundant identified in each of the commercial olive extracts (Table 1). The identities of additional compounds that showed online HPLC-ABTS scavenging activity were unable to be confirmed by LC-MS analysis. Derivatives of elenolic acid (**5**), including the oleosides, in the extract were not phenylpropanoids but rather included a phenylethanoid moiety as a result of esterification from an alternative biosynthetic pathway [48].

Quercetin (**9**) was the most effective flavonoid antioxidant compound in the SOR assay due to its structure, which plays a key role in the scavenging of free radicals. Because of its ready oxidation, it becomes a radical itself, but the resulting unpaired electron is delocalized over the conjugated π-electron system of the molecule, making the quercetin radical too low in energy to be reactive [68]. Additionally, the double bond between C2 and C3 atoms in the chromene (4*H*-1-benzopyran) moiety combined with the carbonyl group at C4 position to become a 4-chromone (4*H*-benzopyran-4-one) increases the ability of quercetin to scavenge free radicals [69].

In contrast, quercetin including all the flavonoids showed poor H_2_O_2_ scavenging activity, which suggests that the flavonoids are the best scavengers of superoxide radicals rather than the hydroxyl radicals. Further investigation of flavonoids against H_2_O_2_ scavenging ability in neuroblastoma cells (SH-SY5Y), shows moderate scavenging properties, and suggests the mechanism of decrease of intracellular ROS induced by H_2_O_2_ was not due to direct H_2_O_2_ scavenging, but rather to scavenging of ROS generated from H_2_O_2_.

The non-flavonoid verbascoside (**6**) was much stronger than oleuropein (**4**), hydroxytyrosol (**3**), or caffeic acid (**1**) against SOR scavenging, H_2_O_2_ radical scavenging and FRAP assay. This may be due to the presence of two catechol moieties in its structure, shows shorter induction times than oleuropein (**4**) in the Rancimat test [70]. In contrast, OLE, which contains oleuropein (**4**) as its major constituent shows the highest SOR scavenging activity and this suggests that the combination of phenolic compounds acting in synergy is more important than any individual biophenol.

In the measurement of total antioxidant capacity by FRAP assay, quercetin (**9**) showed the highest antioxidant activity among flavonoids which agrees with the previously reported studies [71], although the conditions of the analysis were slightly different. It was suggested that *O*-dihydroxy structure in the B-ring and the 3-hydroxy group and 2,3-double bond in the C-ring were responsible for the highest FRAP activity [71]. The olive extract, HTE showed not only the potent H_2_O_2_ scavenging activity, but also the highest FRAP activity, and suggested its main constituent hydroxytyrosol (**3**) in synergistically with the other biophenols, while as alone showed poor to moderate activity. The highest antioxidant and neuroprotective activities of HTE was further verified by the H_2_O_2_-induced toxicity and Cu-induced toxicity in SH-SY5Y cells.

Our results are consistent in terms of potency with studies that have shown olive leaf extracts have higher antioxidant activities than both vitamin C and vitamin E, due to the synergy between the flavonoids, oleuropeosides, and substituted phenols [25]. Indeed, olive biophenols can prevent or minimize oxidative damage processes essentially by scavenging free radical species and/or boosting the endogenous antioxidant system capacity by stimulating, for instance, the synthesis of endogenous antioxidants.

The absorption of the dietary biophenols in olive oil is commonly believed [72] to give rise to insufficient concentrations to account for the activities we report in this communication. Day et al. [73] reported more than 20 quercetin metabolites in human plasma, indicating that flavonols indeed are absorbed, albeit as a range of metabolites. These compounds are also released into the bile and undergo enterohepatic recycling. This process would enhance the absorption of the complete range of olive biophenols possessing catechol moieties. This factor needs to be further considered when discussing the overall bioavailabilities of the olive biophenols.

Despite the misgivings about their *prima facie* bioavailabilities, olive biophenols continue to exhibit the range of reported biological activities, and the use of whole olive leaf and olive leaf extracts have increased rapidly in both the pharmaceutical and food industries as food additives and functional food materials [74,75]. There has been no exact correlation established between the in vitro and in vivo doses of biophenols. However, time-dependent dose-response studies are likely to be way to evaluate the concentrations for specific compounds both in vitro and in vivo.

In general, biophenols exert their antioxidant activity through a chain-breaking mechanism by which the primary antioxidant donates an electron to the free radical present in the systems [76]. Another mechanism involves the removal of ROS/reactive nitrogen species initiators (secondary antioxidants) by quenching these “chain-initiating catalysts” [77]. Furthermore, the antioxidant capacities of biophenols are not limited to only free radical scavenging ability and reducing capacity, but also include the activation of redox transcription factors and up-regulation of genes that induce the expression of antioxidant enzymes [78].

Our study supports the use of olive extracts, which may efficiently achieve health benefits due to the presence of additive and/or synergistic effects of their phytochemicals [79]. Our findings lend support to the dietary and medicinal importance of olive biophenols as an alternative/complementary therapy for the prevention/treatment of neurodegenerative diseases such as Alzheimer’s disease. Nevertheless, further in vivo (transgenic animal) and clinical experiments are recommended before the therapeutic efficacies can be firmly established.

## Figures and Tables

**Table 1:** Identification and online ABTS-scavenging activities of the principal peaks in olive extracts.

Peak	T_R_	λ_max_ (nm)	Identification	ABTS	[M − H]^−^	Extract	References
1	3.6	231, 278	Unknown	Yes	191	OLE	
2	5.1	231, 279	Unknown	Yes	487	OLE	
3	6.1	283	Hydroxytyrosol glucoside	Yes	315	OLE, HTE	[42]
4	6.8	231, 275	Hydroxytyrosol (**3**)	Yes	153	OLE, OFE, HTE, OLP	[42]
5	7.4	232, 333	Caffeic acid glucoside	Yes	339	OLE	[43]
6	8.2	277	Tyrosol (**2**)	No	ND	OLP	[44]
7	8.3	229, 278	Oleuropein aglycone-1	Yes	377	OLE, HTE, OLP	[42]
8	8.5	233	Elenolic acid (**5**)	No	241	OLE	[45]
9	9.1	237	Elenolic acid glucoside	No	403	HTE	[46]
10	11.3	282, 333	Verbascoside (**6**)	Yes	623	OLE, OFE OLP, HTE	[47]
11	12.3	254, 267s, 340	Luteolin-7-*O*-glucoside	Yes	447	OLE, OFE, HTE	[47]
12	13.0	278	Unknown “secoiridoid”	No	581	OLE	
13	14.0	278	Unknown “secoiridoid”	Yes	577	OLE, OFE	
14	14.5	265, 325, 359	Flavonoid glycoside	Yes	447	OLE	
15	14.8	266, 341	Flavonoid glycoside	Yes	461	OLE	
16	15.1	260	Unknown	No	ND	OFE	
17	15.4	237, 280	Oleuropein (**4**)	Yes	539	OFE, OLE	[42]
18	16.0	232	Non-phenolic secoiridoid	No	285	OLP	
19	16.42	234	Oleuroside	Yes	539	OFE, OLE	[47]
20	19.1	287, 290	Oleuropein aglycone-2	Yes	377	HTE	[42]
21	21.2	234, 280	Unknown	No	415	OLE	

T_R_: retention time in minutes; λ_max_: wavelength(s) of maximum absorption in the UV-Vis spectrum; ND: not detected in MS; s: shoulder; OLE: olive leaf extract; OFE: olive fruit extract; HTE: Hydroxytyrosol Extreme; OLP: Olivenol Plus.

**Table 2:** SOR scavenging, H_2_O_2_ scavenging and FRAP value of olive biophenols.

	Biophenols	SOR EC_50_ (μM)	H_2_O_2_ EC_50_ (mM)	FRAP mM TE/g
Non-flavonoids	CA (1)	436.3	1.01	0.830 ± 0.19
	HT (3)	291.4	1.02	0.775 ± 0.16
	OL (4)	258	1.02	0.713 ± 0.18
	VB (6)	119.4	0.66	1.173 ± 0.27
				FRAP mM TE/g
Flavonoids	QU (9)	93.97	NS	1.272 ± 0.29
	RU (10)	143.2	NS	0.957 ± 0.22
	LU (8)	234	NS	1.011 ± 0.23
		SOR EC_50_ (μg/mL)	H_2_O_2_ EC_50_ (μg/mL)	FRAP mg TE/g
Extracts	OLE	1.89	120.6	2.261 ± 0.51
	OFE	6.71	217	1.708 ± 0.39
	HTE	1.98	115.8	2.824 ± 0.64
	OLP	2.46	280.3	1.421 ± 0.32

TE: Trolox^®^ equivalent, NS: not significant, CA: caffeic acid, OL: oleuropein, HT: hydoxytyrosol, VB: verbascoside, QU: quercetin, RU: rutin, LU: luteolin, OLE: olive leaf extract, OFE: olive fruit extract, HTE: hydroxytyrosol extreme, OLP: olivenol plus.

## References

[1] Selkoe D.J. (2001). Alzheimer’s disease: Genes, proteins, and therapy. Physiol. Rev..

[2] Smith M.A., Rottkamp C.A., Nunomura A., Raina A.K., Perry G. (2000). Oxidative stress in Alzheimer’s disease. BBA Mol. Basis Dis..

[3] Pratico D. (2008). Oxidative stress hypothesis in Alzheimer’s disease: A reappraisal. Trends Pharmacol. Sci..

[4] Smith M.A., Harris P.L., Sayre L.M., Perry G. (1997). Iron accumulation in Alzheimer disease is a source of redox-generated free radicals. Proc. Natl. Acad. Sci. USA.

[5] Jomova K., Vondrakova D., Lawson M., Valko M. (2010). Metals, oxidative stress and neurodegenerative disorders. Mol. Cell. Biochem..

[6] Birben E., Sahiner U.M., Sackesen C., Erzurum S., Kalayci O. (2012). Oxidative stress and antioxidant defense. World Allergy Organ. J..

[7] Omar S.H. (2017). Biophenols pharmacology against the amyloidogenic activity in Alzheimer’s disease. Biomed. Pharmacother..

[8] Dai Q., Borenstein A.R., Wu Y., Jackson J.C., Larson E.B. (2006). Fruit and vegetable juices and Alzheimer’s disease: The kame project. Am. J. Med..

[9] Ovaskainen M.L., Torronen R., Koponen J.M., Sinkko H., Hellstrom J., Reinivuo H., Mattila P. (2008). Dietary intake and major food sources of polyphenols in finnish adults. J. Nutr..

[10] Perez-Jimenez J., Fezeu L., Touvier M., Arnault N., Manach C., Hercberg S., Galan P., Scalbert A. (2011). Dietary intake of 337 polyphenols in french adults. Am. J. Clin. Nutr..

[11] Tresserra-Rimbau A., Medina-Remon A., Perez-Jimenez J., Martinez-Gonzalez M.A., Covas M.I., Corella D., Salas-Salvado J., Gomez-Gracia E., Lapetra J., Aros F. (2013). Dietary intake and major food sources of polyphenols in a spanish population at high cardiovascular risk: The predimed study. Nutr. Metab. Cardiovasc. Dis..

[12] Grosso G., Stepaniak U., Topor-Madry R., Szafraniec K., Pajak A. (2014). Estimated dietary intake and major food sources of polyphenols in the polish arm of the hapiee study. Nutrition.

[13] Taguchi C., Fukushima Y., Kishimoto Y., Suzuki-Sugihara N., Saita E., Takahashi Y., Kondo K. (2015). Estimated dietary polyphenol intake and major food and beverage sources among elderly Japanese. Nutrients.

[14] Rothwell J.A., Perez-Jimenez J., Neveu V., Medina-Remon A., M’Hiri N., Garcia-Lobato P., Manach C., Knox C., Eisner R., Wishart D.S. (2013). Phenol-explorer 3.0: A major update of the phenol-explorer database to incorporate data on the effects of food processing on polyphenol content. Database J. Biol. Databases Curation.

[15] Gu L., Kelm M.A., Hammerstone J.F., Beecher G., Holden J., Haytowitz D., Prior R.L. (2003). Screening of foods containing proanthocyanidins and their structural characterization using LC-MS/MS and thiolytic degradation. J. Agric. Food Chem..

[16] Omar S.H., Scott C.J., Hamlin A.S., Obied H.K. (2017). The protective role of plant biophenols in mechanisms of Alzheimer’s disease. J. Nutr. Biochem..

[17] Obied H.K., Prenzler P.D., Omar S.H., Ismael R., Servili M., Esposto S., Taticchi A., Selvaggini R., Urbani S., James C.F. (2012). Chapter six—Pharmacology of olive biophenols. Advances in Molecular Toxicology.

[18] Knoops K.T., de Groot L.C., Kromhout D., Perrin A.E., Moreiras-Varela O., Menotti A., van Staveren W.A. (2004). Mediterranean diet, lifestyle factors, and 10-year mortality in elderly european men and women: The hale project. JAMA.

[19] Sofi F., Macchi C., Abbate R., Gensini G.F., Casini A. (2013). Mediterranean diet and health. Biofactors.

[20] Martinez-Lapiscina E.H., Clavero P., Toledo E., San Julian B., Sanchez-Tainta A., Corella D., Lamuela-Raventos R.M., Martinez J.A., Martinez-Gonzalez M.A. (2013). Virgin olive oil supplementation and long-term cognition: The predimed-navarra randomized, trial. J. Nutr. Health Aging.

[21] Kountouri A.M., Mylona A., Kaliora A.C., Andrikopoulos N.K. (2007). Bioavailability of the phenolic compounds of the fruits (drupes) of olea europaea (olives): Impact on plasma antioxidant status in humans. Phytomedicine.

[22] Omar S.H. (2010). Oleuropein in olive and its pharmacological effects. Sci. Pharm..

[23] Bianco A., Uccella N. (2000). Biophenolic components of olives. Food Res. Int..

[24] Le Tutour B., Guedon D. (1992). Antioxidative activities of olea europaea leaves and related phenolic compounds. Phytochemistry.

[25] Benavente-Garcia O., Castillo J., Lorente J., Ortuño A., Del Rio J.A. (2000). Antioxidant activity of phenolics extracted from *Olea europaea* L. Leaves. Food Chem..

[26] Casaburi I., Puoci F., Chimento A., Sirianni R., Ruggiero C., Avena P., Pezzi V. (2013). Potential of olive oil phenols as chemopreventive and therapeutic agents against cancer: A review of in vitro studies. Mol. Nutr. Food Res..

[27] Obied H.K., Prenzler P.D., Konczak I., Rehman A.U., Robards K. (2009). Chemistry and bioactivity of olive biophenols in some antioxidant and antiproliferative in vitro bioassays. Chem. Res. Toxicol..

[28] Owen R.W., Giacosa A., Hull W.E., Haubner R., Spiegelhalder B., Bartsch H. (2000). The antioxidant/anticancer potential of phenolic compounds isolated from olive oil. Eur. J. Cancer.

[29] Obied H.K., Allen M.S., Bedgood D.R., Prenzler P.D., Robards K. (2005). Investigation of australian olive mill waste for recovery of biophenols. J. Agric. Food Chem..

[30] Zhishen J., Mengcheng T., Jianming W. (1999). The determination of flavonoid contents in mulberry and their scavenging effects on superoxide radicals. Food Chem..

[31] Kamran M., Hamlin A.S., Scott C.J., Obied H.K. (2015). Drying at high temperature for a short time maximizes the recovery of olive leaf biophenols. Ind. Crops Prod..

[32] Benzie I.F., Strain J.J. (1996). The ferric reducing ability of plasma (frap) as a measure of “antioxidant power”: The frap assay. Anal. Biochem..

[33] Xiao Z., Huang C., Wu J., Sun L., Hao W., Leung L.K., Huang J. (2013). The neuroprotective effects of ipriflavone against H_2_O_2_ and amyloid beta induced toxicity in human neuroblastoma SH-SY5Y cells. Eur. J. Pharmacol..

[34] Arciello M., Rotilio G., Rossi L. (2005). Copper-dependent toxicity in SH-SY5Y neuroblastoma cells involves mitochondrial damage. Biochem. Biophys. Res. Commun..

[35] Shi C., Zhao L., Zhu B., Li Q., Yew D.T., Yao Z., Xu J. (2009). Protective effects of ginkgo biloba extract (egb761) and its constituents quercetin and ginkgolide B against β-amyloid peptide-induced toxicity in SH-SY5Y cells. Chem. Biol. Interact..

[36] Chetsawang J., Govitrapong P., Chetsawang B. (2010). Hydrogen peroxide toxicity induces ras signaling in human neuroblastoma SH-SY5Y cultured cells. J. Biomed. Biotechnol..

[37] Mylonaki S., Kiassos E., Makris D.P., Kefalas P. (2008). Optimisation of the extraction of olive (*Olea europaea*) leaf phenolics using water/ethanol-based solvent systems and response surface methodology. Anal. Bioanal. Chem..

[38] Cardoso S.M., Guyot S., Marnet N., Lopes-da-Silva J.A., Renard C.M.G.C., Coimbra M.A. (2005). Characterisation of phenolic extracts from olive pulp and olive pomace by electrospray mass spectrometry. J. Sci. Food Agric..

[39] Hayes J.E., Allen P., Brunton N., O’Grady M.N., Kerry J.P. (2011). Phenolic composition and in vitro antioxidant capacity of four commercial phytochemical products: Olive leaf extract (*Olea europaea* L.), lutein, sesamol and ellagic acid. Food Chem..

[40] Niaounakis M., Halvadakis C.P. (2006). Olive Processing Waste Management: Literature Review and Patent Survey.

[41] Ryan D., Robards K., Lavee S. (1999). Determination of phenolic compounds in olives by reversed-phase chromatography and mass spectrometry. J. Chromatogr. A.

[42] Kontogianni V.G., Charisiadis P., Margianni E., Lamari F.N., Gerothanassis I.P., Tzakos A.G. (2013). Olive leaf extracts are a natural source of advanced glycation end product inhibitors. J. Med. Food.

[43] Chen H.J., Inbaraj B.S., Chen B.H. (2012). Determination of phenolic acids and flavonoids in Taraxacum formosanum Kitam by liquid chromatography-tandem mass spectrometry coupled with a post-column derivatization technique. Int. J. Mol. Sci..

[44] Savarese M., De Marco E., Sacchi R. (2007). Characterization of phenolic extracts from olives (*Olea europaea* cv. Pisciottana) by electrospray ionization mass spectrometry. Food Chem..

[45] Hamden K., Allouche N., Damak M., Elfeki A. (2009). Hypoglycemic and antioxidant effects of phenolic extracts and purified hydroxytyrosol from olive mill waste in vitro and in rats. Chem. Biol. Interact..

[46] Ryan D., Antolovich M., Herlt T., Prenzler P.D., Lavee S., Robards K. (2002). Identification of phenolic compounds in tissues of the novel olive cultivar hardy’s mammoth. J. Agric. Food Chem..

[47] Herrero M., Temirzoda T.N., Segura-Carretero A., Quirantes R., Plaza M., Ibanez E. (2011). New possibilities for the valorization of olive oil by-products. J. Chromatogr. A.

[48] Ryan D., Antolovich M., Prenzler P., Robards K., Lavee S. (2002). Biotransformations of phenolic compounds in *Olea europaea* L.. Sci Hort.

[49] Servili M., Baldioli M., Selvaggini R., Macchioni A., Montedoro G. (1999). Phenolic compounds of olive fruit: One- and two-dimensional nuclear magnetic resonance characterization of nuzhenide and its distribution in the constitutive parts of fruit. J. Agric. Food Chem..

[50] Jovanovic S.V., Steenken S., Tosic M., Marjanovic B., Simic M.G. (1994). Flavonoids as antioxidants. J. Am. Chem. Soc..

[51] D’Imperio M., Cardinali A., D’Antuono I., Linsalata V., Minervini F., Redan B.W., Ferruzzi M.G. (2014). Stability–activity of verbascoside, a known antioxidant compound, at different ph conditions. Food Res. Int..

[52] Cai W., Chen Y., Xie L., Zhang H., Hou C. (2014). Characterization and density functional theory study of the antioxidant activity of quercetin and its sugar-containing analogues. Eur. Food Res. Technol..

[53] Masuoka N., Matsuda M., Kubo I. (2012). Characterisation of the antioxidant activity of flavonoids. Food Chem..

[54] Son T.G., Camandola S., Mattson M.P. (2008). Hormetic dietary phytochemicals. Neuromol. Med..

[55] Connor J.R., Snyder B.S., Beard J.L., Fine R.E., Mufson E.J. (1992). Regional distribution of iron and iron-regulatory proteins in the brain in aging and Alzheimer’s disease. J. Neurosci. Res..

[56] Zecca L., Youdim M.B., Riederer P., Connor J.R., Crichton R.R. (2004). Iron, brain ageing and neurodegenerative disorders. Nat. Rev. Neurosci..

[57] Yamamoto A., Shin R.W., Hasegawa K., Naiki H., Sato H., Yoshimasu F., Kitamoto T. (2002). Iron (iii) induces aggregation of hyperphosphorylated tau and its reduction to iron (ii) reverses the aggregation: Implications in the formation of neurofibrillary tangles of Alzheimer’s disease. J. Neurochem..

[58] Dairam A., Fogel R., Daya S., Limson J.L. (2008). Antioxidant and iron-binding properties of curcumin, capsaicin, and S-allylcysteine reduce oxidative stress in rat brain homogenate. J. Agric. Food Chem..

[59] Miller E.W., Dickinson B.C., Chang C.J. (2010). Aquaporin-3 mediates hydrogen peroxide uptake to regulate downstream intracellular signaling. Proc. Natl. Acad. Sci. USA.

[60] Bindoli A., Fukuto J.M., Forman H.J. (2008). Thiol chemistry in peroxidase catalysis and redox signaling. Antioxid. Redox Signal..

[61] Milton N.G. (2004). Role of hydrogen peroxide in the aetiology of Alzheimer’s disease: Implications for treatment. Drugs Aging.

[62] Shen C., Chen Y., Liu H., Zhang K., Zhang T., Lin A., Jing N. (2008). Hydrogen peroxide promotes abeta production through jnk-dependent activation of gamma-secretase. J. Biol. Chem..

[63] Jo D.G., Arumugam T.V., Woo H.N., Park J.S., Tang S.C., Mughal M., Hyun D.H., Park J.H., Choi Y.H., Gwon A.R. (2010). Evidence that γ-secretase mediates oxidative stress-induced β-secretase expression in Alzheimer’s disease. Neurobiol. Aging.

[64] Arnal N., de Alaniz M.J., Marra C.A. (2012). Cytotoxic effects of copper overload on human-derived lung and liver cells in culture. Biochim. Biophys. Acta.

[65] Watt N.T., Hooper N.M. (2001). The response of neurones and glial cells to elevated copper. Brain Res. Bull..

[66] Deane R., Bell R.D., Sagare A., Zlokovic B.V. (2009). Clearance of amyloid-β peptide across the blood-brain barrier: Implication for therapies in Alzheimer’s disease. CNS Neurol. Disord. Drug Targets.

[67] Bandaruk Y., Mukai R., Terao J. (2014). Cellular uptake of quercetin and luteolin and their effects on monoamine oxidase-α in human neuroblastoma SH-SY5Y cells. Toxicol. Rep..

[68] Mariani C., Braca A., Vitalini S., De Tommasi N., Visioli F., Fico G. (2008). Flavonoid characterization and in vitro antioxidant activity of *Aconitum anthora* L. (ranunculaceae). Phytochemistry.

[69] Brown J.E., Khodr H., Hider R.C., Rice-Evans C.A. (1998). Structural dependence of flavonoid interactions with Cu^2+^ ions: Implications for their antioxidant properties. Biochem. J..

[70] Artajo L.S., Romero M.P., Morello J.R., Motilva M.J. (2006). Enrichment of refined olive oil with phenolic compounds: Evaluation of their antioxidant activity and their effect on the bitter index. J. Agric. Food Chem..

[71] Firuzi O., Lacanna A., Petrucci R., Marrosu G., Saso L. (2005). Evaluation of the antioxidant activity of flavonoids by “ferric reducing antioxidant power” assay and cyclic voltammetry. Biochim. Biophys. Acta.

[72] Vissers M.N., Zock P.L., Katan M.B. (2004). Bioavailability and antioxidant effects of olive oil phenols in humans: A review. Eur. J. Clin. Nutr..

[73] Day A.J., Mellon F., Barron D., Sarrazin G., Morgan M.R., Williamson G. (2001). Human metabolism of dietary flavonoids: Identification of plasma metabolites of quercetin. Free Radic. Res..

[74] Delgado-Pertiñez M., Gómez-Cabrera A., Garrido A. (2000). Predicting the nutritive value of the olive leaf (*Olea europaea*): Digestibility and chemical composition and in vitro studies. Anim. Feed Sci. Technol..

[75] Omar S.H. (2018). Chapter 4—Biophenols: Impacts and prospects in anti-Alzheimer drug discovery a2-brahmachari, goutam. Discovery and Development of Neuroprotective Agents from Natural Products.

[76] Lindenmeier M., Burkon A., Somoza V. (2007). A novel method to measure both the reductive and the radical scavenging activity in a linoleic acid model system. Mol. Nutr. Food Res..

[77] Young I.S., Woodside J.V. (2001). Antioxidants in health and disease. J. Clin. Pathol..

[78] Liang N., Kitts D.D. (2014). Antioxidant property of coffee components: Assessment of methods that define mechanisms of action. Molecules.

[79] Pereira A.P., Ferreira I.C., Marcelino F., Valentao P., Andrade P.B., Seabra R., Estevinho L., Bento A., Pereira J.A. (2007). Phenolic compounds and antimicrobial activity of olive (*Olea europaea* L. Cv. Cobrancosa) leaves. Molecules.

